# Watching gene expression in color

**DOI:** 10.7554/eLife.49414

**Published:** 2019-07-26

**Authors:** Julie H Simpson

**Affiliations:** 1 Department of Molecular, Cellular, and Developmental BiologyUniversity of California, Santa BarbaraSanta BarbaraUnited States; 2 Neuroscience Research InstituteUniversity of California, Santa BarbaraSanta BarbaraUnited States

**Keywords:** transcriptional dynamics, fluorescent reporter, transcriptional timer, *D. melanogaster*

## Abstract

A combination of two fluorescent proteins with different half-lives allows gene expression to be followed with improved time resolution.

**Related research article** He L, Binari R, Huang J, Falo-Sanjuan J, Perrimon N. 2019. In vivo study of gene expression with an enhanced dual-color fluorescent transcriptional timer. *eLife* **8**:e46181. doi: 10.7554/eLife.46181

Cells are dynamic places and the levels of gene products – RNA molecules and proteins – inside a given cell change over time. Moreover, different types of cells contain different constellations of RNA molecules and proteins. These aspects of cell identity are controlled by gene expression – the process by which genes are transcribed to form messenger RNA (mRNA) molecules, some of which are then translated to produce proteins.

Many techniques are available to study gene expression in cells. Single-cell RNA sequencing provides a global view of the transcriptional profiles of cells (Bates et al., 2019). In fixed tissue samples, in situ hybridization can be used to detect mRNA molecules, while immunohistochemistry techniques involving antibodies can detect proteins. 'Enhancer bashing' methods have been used to identify the regulatory elements that govern when and where particular genes are expressed (Borok et al., 2010). 'Enhancer traps' and 'protein traps' rely on reporters – this is, genes that produce an easily detectable protein – to provide information on the expression of neighboring genes of interest (St Johnston, 2002). 

Fluorescent proteins are widely used as reporters for gene expression. When illuminated with certain wavelengths of light, these proteins emit fluorescent light of a characteristic color that can then be detected. There are also fluorescent proteins that change color over time or when exposed to light of a specific wavelength (Lin and Tsien, 2010). Most cells do not produce their own fluorescent proteins, so DNA constructs containing the sequence for the fluorescent protein have to be introduced. The insertion can happen either at the normal locus of the gene or at a defined landing site. Fluorescent proteins placed under the regulatory sequences of a gene of interest can then be used to report on the expression of that gene.

Previously, relationships between cells could be detected by inducing dividing cells to express one of several fluorescent proteins at specific time-points during development (Lee and Luo, 1999; Cachero and Jefferis, 2011). Now, in eLife, Li He, Norbert Perrimon and colleagues at Harvard Medical School, Chongqing University and Tufts University report how they have combined two fluorescent proteins with different half-lives to make a reporter (which they call a transcriptional timer or TransTimer) that can be used to explore the dynamics of gene expression in fruit flies (He et al., 2019).

A bright, fast-folding version of green fluorescent protein was selected and modified to speed up its translation. First, He et al. optimized protein synthesis by ensuring that the most common triplet codons available in the fly were used to make the protein. Next, sequences were added to make more of the fluorescent protein by increasing translation initiation, increasing transport of mRNA to the cytoplasm, and efficiently poly-adenylating the mRNA so it could be found by the translation machinery (Pfeiffer et al., 2012). Last, they added a sequence to target the protein for degradation in order to ensure a short half-life. This kind of careful protein engineering – with emphasis on temporal precision of the off switch – is similar to that which led to dramatic improvements in the calcium sensors that can report action potentials in neurons (Dana et al., 2019).

In vitro and in vivo tests showed that these modifications resulted in a rapid increase and decrease of the fluorescent signal, with green fluorescence being detected within 10 minutes and disappearing inside of two hours. A slow-folding, stable red fluorescent protein which can be detected after 1.5 hours, and which lasts for more than 20 hours, was added to generate the TransTimer reporter.

Due to the different folding and degradation times of the two proteins in the TransTimer, the onset of the green fluorescence is fast, followed by a more gradual rise in red fluorescence. If a gene is stably expressed, both green and red fluorescence will be detected. On the other hand, if a gene is dynamically expressed, after an initial period during which both colors can be detected, only red fluorescence will be observed. These fluorescent proteins are bright enough that the changes in color can be watched in living tissue, and potentially tracked in experiments involving long-term imaging of developing embryos (Royer et al., 2016).

He et al. demonstrated some of the applications of the TransTimer in the fruit fly. First, they showed that it can be used to detect short bursts of gene expression in fixed tissues (by measuring the proportions of green to red fluorescent proteins), and that it can help identify new genes with dynamic regulation through a TransTimer enhancer trap. Next it was shown that the TransTimer can be used to follow the gene expression history of different cell types. For example, neurons are sequentially produced by neuroblast stem cells, and key genes in this differentiation are transiently expressed. With the TransTimer, cells that currently express a gene will fluoresce both green and red, while cells that have already switched it off will only emit red fluorescence. In tissues like the eye or wing disc, this resulted in a leading edge of green/red cells followed by a wave of red ones.

The TransTimer could also have other applications in flies. First, it could allow the study of gene expression in dynamic processes such as circadian rhythms and metamorphosis. Second, it could provide real-time information about gene expression during cell fate assignments, complementing mRNA sequencing data, which only provides a snapshot of this process (Bates et al., 2019). Finally, the TransTimer's ability to record gene expression history could be used to answer mechanistic questions about the gene expression cascades that establish neuron identity in developing *Drosophila* brains (Doe, 2017). It should also be possible to adapt the TransTimer for use in other organisms.

The regulation of gene expression is complex and many questions remain. By showing when, where and how much a gene is expressed, reporters such as the TransTimer will be valuable tools in our efforts to better understand this process.
